# Supplementary material for: Dynamic natural components and morphological changes in nonculprit subclinical atherosclerosis in patients with acute coronary syndrome and mild chronic kidney disease at the 1-year follow-up and clinical significance at the 5-year follow-up
Source: PLoS One. 2024 May 31;19(5):e0302547. doi: 10.1371/journal.pone.0302547 (PMC11142449; doi:10.1371/journal.pone.0302547)
Supplement: Attachment — Submitted filename: Response to Reviewers.docx [file pone.0302547.s001.docx]

Please include the following items when submitting your revised manuscript:

A rebuttal letter that responds to each point raised by the academic editor and reviewer(s). You should upload this letter as a separate file labeled 'Response to Reviewers'.

A marked-up copy of your manuscript that highlights changes made to the original version. You should upload this as a separate file labeled 'Revised Manuscript with Track Changes'.

An unmarked version of your revised paper without tracked changes. You should upload this as a separate file labeled 'Manuscript'.

If you would like to make changes to your financial disclosure, please include your updated statement in your cover letter. Guidelines for resubmitting your figure files are available below the reviewer comments at the end of this letter.

If applicable, we recommend that you deposit your laboratory protocols in protocols.io to enhance the reproducibility of your results. Protocols.io assigns your protocol its own identifier (DOI) so that it can be cited independently in the future. For instructions see: https://journals.plos.org/plosone/s/submission-guidelines#loc-laboratory-protocols. Additionally, PLOS ONE offers an option for publishing peer-reviewed Lab Protocol articles, which describe protocols hosted on protocols.io. Read more information on sharing protocols at https://plos.org/protocols?utm_medium=editorial-email&utm_source=authorletters&utm_campaign=protocols.

We look forward to receiving your revised manuscript.

Kind regards,

Hean Teik Ong

Academic Editor

PLOS ONE

Journal Requirements:

1. When submitting your revision, we need you to address these additional requirements.

Please ensure that your manuscript meets PLOS ONE's style requirements, including those for file naming. The PLOS ONE style templates can be found at

https://journals.plos.org/plosone/s/file?id=wjVg/PLOSOne_formatting_sample_main_body.pdf and

https://journals.plos.org/plosone/s/file?id=ba62/PLOSOne_formatting_sample_title_authors_affiliations.pdf.

**A: thanks for your recommendation, and we have changed the manuscript style according to the PLOS ONE style requirements.**

2. We note that the grant information you provided in the ‘Funding Information’ and ‘Financial Disclosure’ sections do not match.

When you resubmit, please ensure that you provide the correct grant numbers for the awards you received for your study in the ‘Funding Information’ section.

**A: Thank you for your criticism and correction. We did not express it clearly in the original manuscript, but we have corrected it in the revised draft.**

3. Thank you for stating the following in your Competing Interests section:

[None].

Please complete your Competing Interests on the online submission form to state any Competing Interests. If you have no competing interests, please state "The authors have declared that no competing interests exist.", as detailed online in our guide for authors at http://journals.plos.org/plosone/s/submit-now

This information should be included in your cover letter; we will change the online submission form on your behalf.

**A: Yes, we corrected it.**

4. We note that you have indicated that there are restrictions to data sharing for this study. PLOS only allows data to be available upon request if there are legal or ethical restrictions on sharing data publicly. For more information on unacceptable data access restrictions, please see http://journals.plos.org/plosone/s/data-availability#loc-unacceptable-data-access-restrictions.

Before we proceed with your manuscript, please address the following prompts:

a) If there are ethical or legal restrictions on sharing a de-identified data set, please explain them in detail (e.g., data contain potentially identifying or sensitive patient information, data are owned by a third-party organization, etc.) and who has imposed them (e.g., a Research Ethics Committee or Institutional Review Board, etc.). Please also provide contact information for a data access committee, ethics committee, or other institutional body to which data requests may be sent.

b) If there are no restrictions, please upload the minimal anonymized data set necessary to replicate your study findings to a stable, public repository and provide us with the relevant URLs, DOIs, or accession numbers. For a list of recommended repositories, please see https://journals.plos.org/plosone/s/recommended-repositories. You also have the option of uploading the data as Supporting Information files, but we would recommend depositing data directly to a data repository if possible.

We will update your Data Availability statement on your behalf to reflect the information you provide.

**A: We have uploaded the data as supporting information files.**

5. Your ethics statement should only appear in the Methods section of your manuscript. If your ethics statement is written in any section besides the Methods, please move it to the Methods section and delete it from any other section. Please ensure that your ethics statement is included in your manuscript, as the ethics statement entered into the online submission form will not be published alongside your manuscript.

**A: Yes, we corrected it.**

6. We note that Figure 2 and 3 in your submission contain copyrighted images. All PLOS content is published under the Creative Commons Attribution License (CC BY 4.0), which means that the manuscript, images, and Supporting Information files will be freely available online, and any third party is permitted to access, download, copy, distribute, and use these materials in any way, even commercially, with proper attribution. For more information, see our copyright guidelines: http://journals.plos.org/plosone/s/licenses-and-copyright.

We require you to either (1) present written permission from the copyright holder to publish these figures specifically under the CC BY 4.0 license, or (2) remove the figures from your submission:

a. You may seek permission from the original copyright holder of Figure 2 and 3 to publish the content specifically under the CC BY 4.0 license.

We recommend that you contact the original copyright holder with the Content Permission Form (http://journals.plos.org/plosone/s/file?id=7c09/content-permission-form.pdf) and the following text:

“I request permission for the open-access journal PLOS ONE to publish XXX under the Creative Commons Attribution License (CCAL) CC BY 4.0 (http://creativecommons.org/licenses/by/4.0/). Please be aware that this license allows unrestricted use and distribution, even commercially, by third parties. Please reply and provide explicit written permission to publish XXX under a CC BY license and complete the attached form.”

Please upload the completed Content Permission Form or other proof of granted permissions as an ""Other"" file with your submission.

In the figure caption of the copyrighted figure, please include the following text: “Reprinted from [ref] under a CC BY license, with permission from [name of publisher], original copyright [original copyright year].”

b. If you are unable to obtain permission from the original copyright holder to publish these figures under the CC BY 4.0 license or if the copyright holder’s requirements are incompatible with the CC BY 4.0 license, please either i) remove the figure or ii) supply a replacement figure that complies with the CC BY 4.0 license. Please check copyright information on all replacement figures and update the figure caption with source information. If applicable, please specify in the figure caption text when a figure is similar but not identical to the original image and is therefore for illustrative purposes only.

**A: Figures 2 and 3 in this article are original drawings of our own creation and are not copied from other articles or books. Figure 2 was one of the optical coherence tomography (OCT) images of our observational cases analyzed by the optical flow ratio (OFR) software. Figure 3 shows the original OCT cross section image on the left, and the measurement method diagram on the right, that is, the measurement method adopted in our study, which is the original measurement method of our working group.**

7. Please include your tables as part of your main manuscript and remove the individual files. Please note that supplementary tables (should remain/ be uploaded) as separate ""supporting information"" files".

**A: Yes, we have corrected it.**

8. Please review your reference list to ensure that it is complete and correct. If you have cited papers that have been retracted, please include the rationale for doing so in the manuscript text, or remove these references and replace them with relevant current references. Any changes to the reference list should be mentioned in the rebuttal letter that accompanies your revised manuscript. If you need to cite a retracted article, indicate the article’s retracted status in the References list and also include a citation and full reference for the retraction notice.

**A: We have rechecked all references, and some reference list was corrected according to reviews recommendation in our cite in the revised manuscript.**

[Note: HTML markup is below. Please do not edit.]

**Reviewers' comments:**

Reviewer's Responses to Questions

Comments to the Author

1. Is the manuscript technically sound, and do the data support the conclusions?

The manuscript must describe a technically sound piece of scientific research with data that supports the conclusions. Experiments must have been conducted rigorously, with appropriate controls, replication, and sample sizes. The conclusions must be drawn appropriately based on the data presented.

Reviewer #1: Yes

Reviewer #2: Yes

2. Has the statistical analysis been performed appropriately and rigorously?

Reviewer #1: Yes

Reviewer #2: Yes

3. Have the authors made all data underlying the findings in their manuscript fully available?

The PLOS Data policy requires authors to make all data underlying the findings described in their manuscript fully available without restriction, with rare exception (please refer to the Data Availability Statement in the manuscript PDF file). The data should be provided as part of the manuscript or its supporting information, or deposited to a public repository. For example, in addition to summary statistics, the data points behind means, medians and variance measures should be available. If there are restrictions on publicly sharing data—e.g. participant privacy or use of data from a third party—those must be specified.

Reviewer #1: Yes

Reviewer #2: Yes

4. Is the manuscript presented in an intelligible fashion and written in standard English?

PLOS ONE does not copyedit accepted manuscripts, so the language in submitted articles must be clear, correct, and unambiguous. Any typographical or grammatical errors should be corrected at revision, so please note any specific errors here.

Reviewer #1: Yes

Reviewer #2: Yes

5. Review Comments to the Author

Please use the space provided to explain your answers to the questions above. You may also include additional comments for the author, including concerns about dual publication, research ethics, or publication ethics. (Please upload your review as an attachment if it exceeds 20,000 characters)

**Reviewer #1:**

**1.** **Classification of CKD should use** **the CKD-Epi 2021 equation rather than the MDRD equation**

**A:** Thank you for your kind recommendation, we have corrected the old MDRD equation to the CKD-EPI 2021 equation based on two important references (Chronic Kidney Disease Epidemiology Collaboration. New Creatinine- and Cystatin C-Based Equations to Estimate GFR without Race. N Engl J Med. 2021 Nov 4;385(19):1737-1749. doi: 10.1056/NEJMoa2102953. Epub 2021 Sep 23. And National Kidney Foundation Laboratory Engagement Working Group Recommendations for Implementing the CKD-EPI 2021 Race-Free Equations for Estimated Glomerular Filtration Rate: Practical Guidance for Clinical Laboratories Clin Chem. 2022 Mar 31;68(4):511-520. doi: 10.1093/clinchem/hvab278). And at the meantime, we modified the corresponding references in the revised manuscript.

**2. Has Tables 1-3 been submitted? Does not seem to be included in the manuscript.**

**A:** It was our fault. We uploaded the tables into the “other” column before, and now we have corrected it and inserted the Table1-3 into the revised manuscript.

**3. The change in renal function described on page 17 from line 9 to 16 is confusing and should be summarized.**

**A:** Thank you for your kind recommendation. We have revised our wording as recommended by the Guide as follows (Staging renal function are performed as recommended in the guidelines of KDIGO criteria, which defines that G1 as increased (eGFR > 105 mL/(min×1.73 m^2^)) or optimal (105 mL/(min×1.73 m^2^) >eGFR >90 mL/(min×1.73 m^2^)) renal function, G2 as mild CKD (89 mL/(min×1.73 m^2^) >eGFR >60 mL/(min×1.73 m^2^)), G3a as mild-moderate CKD (59 mL/(min×1.73 m^2^) >eGFR >45 mL/(min×1.73 m^2^)), G3b as moderate-severe CKD (44 mL/(min×1.73 m^2^) >eGFR >30 mL/(min×1.73 m^2^)), G4 as severe CKD (29 mL/(min×1.73 m^2^) >eGFR >15 mL/(min×1.73 m^2^)), and G5 as kidney failure (eGFR < 15 mL/(min×1.73 m^2^)).) and added it to the revised manuscript. And meanwhile, we modified the corresponding references in the revised manuscript.

**4. Was there an increase in calcification in the media?**

**A:** We totally agree with your opinion, but since intracoronary imaging of OCT cannot clearly distinguish between intimal calcification and media calcification (calcification lesions are often distributed in both intima and media at the same time, and there is no clear boundary during the period), in this study, we can only observe the direction of calcification growth (whether arc or thickness), and the final conclusion is that the thickness increases significantly, and the protrusion to the lumen side is more significant. However, due to the small amplitude, it can only be detected from the quantitative analysis of OCT, and the quantitative analysis of intimal or media calcification cannot be carried out. **We have added this point into the limitations of our revised manuscript.**

**5. There is a lack of correlation between lesions detected and clinical outcomes**

**A:** The 5-year clinical follow-up showed that there were more nonculprit subclinical atherosclerosis (NSA)-related MACEs in mild CKD than non-CKD (30.8% vs. 5.8%, p=0.045) by Kaplan‒Meier analysis (shown in Fig 4). As shown in the figure, the two curves intersected near the 3-year follow-up. Landmark analysis showed that there was no significant difference in the NSA-related MACEs between the two groups within nearly 3 years after PCI, while the MACEs in mild CKD became significantly more common than those in non-CKD (25.7% vs. 0.7%, p=0.006) during 3 to 5 years (shown in Fig 5). Further analysis of the components of MACEs related to NSA revealed that the whole difference was due to ischemia-driven revascularization (24.6% vs. 5.8%, p=0.365), cardiac death (7.4% vs. 0%, p=0.023) and myocardial infarction (3.8% vs. 1.1%, p=0.40) in mild CKD compared with non-CKD.

The above contents have been described in the conclusion of the original manuscript, but due to the relatively small number of cases, this theory needs to be verified by further clinical studies. We also re-emphasize this point at the end of the manuscript.

**Reviewer #2: Need to rephrase these sentences in Page 22**

**“Although many factors are associated with coronary artery calcification progression, such as old age, smoking, diabetes and CKD[CKD [42,43], the current theory is that intimal calcification is related to atherosclerosis, and inflammation contributes, while the characteristics of coronary artery calcification in patients with CKD (especially stages 4-5) are characterized by calcification of the medial layer[39,40,44]. However, atherosclerotic processes dominate in patients with early stages of CKD (stages 1-2)[2) [45,46]. The actual situation is that in our daily practice, the intima and media of the coronary artery are both calcified in CKD patients, which may be related to the participation of multiple factors[34]. ”**

**A:** Thank you for your kind recommendation. We have corrected it as follow in the revised manuscript:

Although many factors are associated with the progression of coronary artery calcification, such as age, smoking, diabetes, and CKD [43, 44], some studies have demonstrated that media coronary calcification was correlated with severe CKD or kidney failure (G4-5) patients [40, 41, 45], however, other studies in mild CKD patients or patients with normal renal function (G1-2) have shown that atherosclerosis was dominant and more manifested by intimal calcification [46, 47], in which most of these studies used computerized tomography images for analysis [41, 42, 44], and a few of them used intracoronary imaging such as OCT [43, 48, 49]. Our study found that patients with mild CKD may have calcification growth in both intima and media of coronary arteries, which may be related to the involvement of multiple factors[35].

6. PLOS authors have the option to publish the peer review history of their article (what does this mean?). If published, this will include your full peer review and any attached files.

If you choose “no”, your identity will remain anonymous but your review may still be made public.

Do you want your identity to be public for this peer review? For information about this choice, including consent withdrawal, please see our Privacy Policy.

Reviewer #1: No

Reviewer #2: Yes: Lee Boon Chye

[NOTE: If reviewer comments were submitted as an attachment file, they will be attached to this email and accessible via the submission site. Please log into your account, locate the manuscript record, and check for the action link "View Attachments". If this link does not appear, there are no attachment files.]

While revising your submission, please upload your figure files to the Preflight Analysis and Conversion Engine (PACE) digital diagnostic tool, https://pacev2.apexcovantage.com/. PACE helps ensure that figures meet PLOS requirements. To use PACE, you must first register as a user. Registration is free. Then, login and navigate to the UPLOAD tab, where you will find detailed instructions on how to use the tool. If you encounter any issues or have any questions when using PACE, please email PLOS at figures@plos.org. Please note that Supporting Information files do not need this step.
